# Preparation of High Density Polyethylene/Waste Polyurethane Blends Compatibilized with Polyethylene-Graft-Maleic Anhydride by Radiation

**DOI:** 10.3390/ma8041626

**Published:** 2015-04-08

**Authors:** Jong-Seok Park, Youn-Mook Lim, Young-Chang Nho

**Affiliations:** Radiation Research Division for Industry and Environment, Korea Atomic Energy Research Institute, 1266 Sinjeong-dong, Jeongeup-si, Jeollabuk-do 580-185, Korea; E-Mails: ymlim71@kaeri.re.kr (Y.-M.L.); jaspa@hanmail.net (Y.-C.N.)

**Keywords:** waste, polyurethane, radiation grafting, recycling, polyethylene

## Abstract

Polyurethane (PU) is a very popular polymer that is used in a variety of applications due to its good mechanical, thermal, and chemical properties. However, PU recycling has received significant attention due to environmental issues. In this study, we developed a recycling method for waste PU that utilizes the radiation grafting technique. Grafting of waste PU was carried out using a radiation technique with polyethylene-graft-maleic anhydride (PE-g-MA). The PE-g-MA-grafted PU/high density polyethylene (HDPE) composite was prepared by melt-blending at various concentrations (0–10 phr) of PE-g-MA-grafted PU. The composites were characterized using fourier transform infrared spectroscopy (FT-IR), and their surface morphology and thermal/mechanical properties are reported. For 1 phr PU, the PU could be easily introduced to the HDPE during the melt processing in the blender after the radiation-induced grafting of PU with PE-g-MA. PE-g-MA was easily reacted with PU according to the increasing radiation dose and was located at the interface between the PU and the HDPE during the melt processing in the blender, which improved the interfacial interactions and the mechanical properties of the resultant composites. However, the elongation at break for a PU content >2 phr was drastically decreased.

## 1. Introduction

Polyurethane (PU) is a very popular polymer that is used in a variety of applications, such as in vehicles, furniture, and construction materials, due to its good mechanical, thermal, and chemical properties [1,2]. However, PU recycling has received significant attention due to environmental issues. Increasing landfill costs and decreasing landfill space have facilitated considerations of alternative options for the disposal of PU. Thus, there is a need to recycle or modify waste PU into some useful materials [3,4]. PU can be recycled by several methods, such as mechanical recycling, advanced chemical and thermo chemical recycling, energy recovery and product recycling [3]. Among these methods, mechanical recycling is performed by regrinding polyurethane foam into powder, allowing the modified materials to be reused as a filler [3].

Recently, nano fillers, such as nanoclay, nanosilica, carbon nanotubes and grapheme, have been used as commercial fillers for the polymer composite [5]. However, these materials are too expensive for mass production. In addition, the compatibility between the polymer and the filler significantly affects the quality of the composite [5].

PU is added as a reinforcing filler to enhance the toughness and thermal properties of commodity polymers [6]. However, the major disadvantage of incorporating PU into commodity polymers is its compatibility. PU is hydrophilic, whereas most commodity polymers are hydrophobic [7,8].

Surface modification or grafting polymerization is an excellent way to enhance the adhesion of immiscible polymers or the adhesion between the polymer and the filler [5,9]. Radiation-induced grafting, in comparison with classical chemical grafting, provides several advantages such as uniform grafting and room temperature processing, and freedom from the residuals of the initiator or catalyst [4,10,11]. In particular, the radiation technique is able to transfer energy to the solid state to create free radicals [12].

Maleic anhydride-graft-polyolefin has been widely used to improve the interfacial interaction between immiscible polymers [13].

Here, we developed a recycling method for waste PU that utilizes the radiation grafting technique. The grafting of waste PU was carried out using a radiation technique with polyethylene-graft-maleic anhydride (PE-g-MA). The PE-g-MA-grafted PU/high density polyethylene (HDPE) composite was prepared through the melt-blending. Enhanced compatibility was obtained, and the mechanical properties of the composite were determined.

## 2. Experimental

### 2.1. Materials

Commercial grade high-density polyethylene (HDPE; 5305E) was used throughout this study and was supplied by Lotte Chemical Corporation (Yeosu, Korea). Polyurethane (PU) with a yeollowish color was collected from railway waste disposal of Envista, Inc. (Chungju, Korea). Polyethylene-graft-maleic anhydride (PE-g-MA) was purchased from Sigma Aldrich (St. Louis, MO, USA). Dimethylformamide was purchased from Samchun Pure Chemicals Co., Ltd. (Pyeongtaek, Korea). All other chemicals used were of reagent grade and were applied as purchased without further purification.

### 2.2. Radiation-Induced Grafting

The waste PU particles (15 wt%) and PE-g-MA (5 wt%) were immersed in a dimethylformamide (DMF) solution (80 wt%) and mechanically stirred at room temperature until the chemicals were thoroughly dissolved. The prepared mixtures were irradiated using an electron beam accelerator (10 MeV/1 mA, Jeongup site of KAERI, Jeonbuk, Korea). The total absorbed dose ranged from 10 to 70 kGy. Thereafter, to remove the remaining solvent and moisture, the irradiated mixtures were washed 3 times with DI water and dried in a vacuum oven at 60 °C for 24 h.

### 2.3. Preparation the HDPE/PU Composites

The PE-g-MA-grafted PU/HDPE composites were prepared by melt-blending at various concentrations (0–10 phr) of PE-g-MA-grafted PU. The unit “phr” is the abbreviation of parts per on hundred resin, and the base resin used here is HDPE. Melt compounding was carried out using a lab-scale blender (Brabender D-47055, Brabender, Germany) with a screw speed of 50 rpm at 160 °C for 15 min. After blending, the mixtures were prepared using square-shaped sheets via hot-press molding at 180 °C for 10 min.

### 2.4. Characterization of the PU/HDPE Composite

Mechanical properties, such as tensile strength and elongation at break, were measured using a universal testing machine (Model 4210, Instron Engineering Co., Norwood, MA, USA) according to the ASTM Standard D 638 [14]. The measurements were taken at room temperature at a crosshead speed of 50 mm/min.

The radiation-induced grafting of PU onto the HDPE composites was analyzed via FT-IR (Tensor 37, Bruker, Billerica, MA, USA).

The surface morphologies of the PU/HDPE composites were observed using a scanning electron microscope (SEM, Hitachi, S-4700, Tokyo, Japan).

The static water contact angles were measured according to the sessile drop method using a Phoenix 300 contact angle analyzer (Surface Electro Optics Co., Suwon, Korea). Regarding the water contact angle, at least five specimens were tested, and the average value was taken.

The storage modulus was measured using a dynamic mechanical analyzer (DMA, TA Instrument Q800, New Castle, NH, USA). The temperature was increased from room temperature to 130 °C at a heating rate of 5 °C/min, and the oscillation frequency was 1 Hz.

The melting behavior and the crystallinity of the composites was determined based on differential scanning calorimetry (DSC) (TA Instrument Q100, New Castle, NH, USA) from 30 to 180 °C at a heating rate of 10 °C/min under nitrogen gas. The degree of crystallinity (*X_c_*) can be calculated by the following equation.

(1) $$Xc(\%)=\frac{\Delta H_{m}}{\Delta H_{m}^{o}}\times 100$$

where $\Delta H_{m}^{o}$ = 290 J/g is the fusion enthalpy for a totally crystalline polymer and Δ*H_m_* is the fusion enthalpy calculated from the area of the endothermic melting peak.

## 3. Results and Discussion

It is well known that PU/HDPE composites exhibit poor mechanical properties, such as tensile strength and elongation at break, due to their incompatibility [15].

To fabricate a good composite, improved compatibility between HDPE and PU is very important. In this study, the effect of the radiation grafting on PU/HDPE composites for developing a composite with better properties was studied.

**Figure 1:**
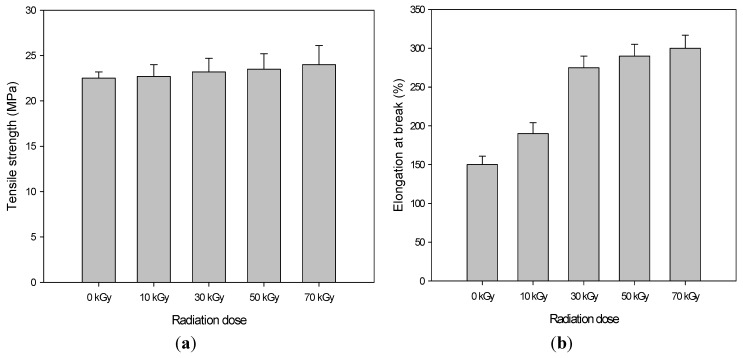
(**a**) Tensile strength and (**b**) elongation at break polyurethane (PU) (1 phr)/high density polyethylene (HDPE) composite at various absorption doses.

Figure 1 shows the mechanical properties, including tensile strength and elongation at break of the PU/HDPE composites at various absorption doses. During this experiment, the content of PU was 1 phr. The mechanical properties of the samples were tested at least five times, and the average value at the maximum point was recorded.

The tensile strength slightly increased with increasing the radiation doses. As shown in Figure 1a, at an irradiation doe of 70 kGy, the tensile strength was 24 MPa. In addition, the elongation at break drastically increased with increasing absorbed doses. When the absorbed dose was 70 kGy, the elongation at break of the PU/HDPE composites reached a maximum average value of 300%, which is approximately twofold greater than that of non-irradiated PU/HDPE composite (150%). This result indicates a significant improvement in the interfacial state between the HDPE and the PU after the radiation-induced grafting of PU.

**Figure 2:**
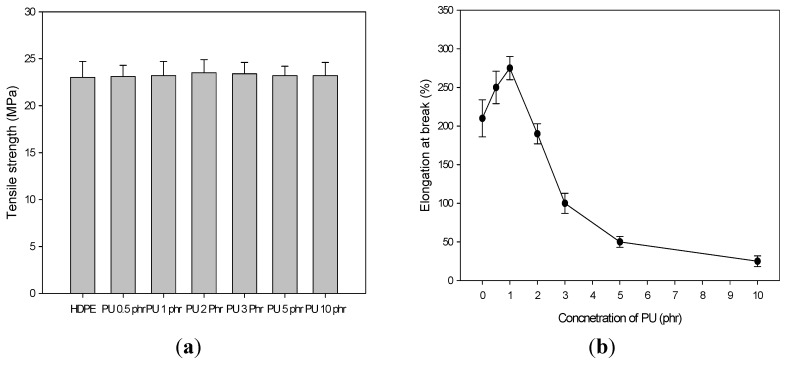
(**a**) Tensile strength and (**b**) elongation at break PU/HDPE composite at various content of PU; absorption dose is 30 kGy.

Figure 2 shows the tensile strength and elongation at break of the PU/HDPE composites for various contents of PU. In this experiment, the radiation dose was 30 kGy. No changes in the tensile strength were observed in the PU/HDPE composites with increasing concentrations of PU. However, the elongation at break of the PU/HDPE composites increased with increasing contents of PU up to 1 phr. When the content of PU was 1 phr, the maximum elongation at break was approximately 280%. However, the elongation at break with a PU content >2 phr drastically decreased. When the content of PU was 10 phr, the maximum elongation at break exhibited the lowest value of approximately 30% and was approximately nine times lower than that of the composite containing 1 phr PU. This result suggests that when the content of PU in the HDPE composite is >2 phr, the compatibility between the PU and the HDPE are drastically deteriorated, and incompatibility is observed even though the PU and PE-g-MA undergo electron beam irradiation.

FT-IR, SEM and water contact angle measurements were carried out to assess whether the PU was grafted to the surface of PE-g-MA as a compatibilizer for HDPE.

**Figure 3:**
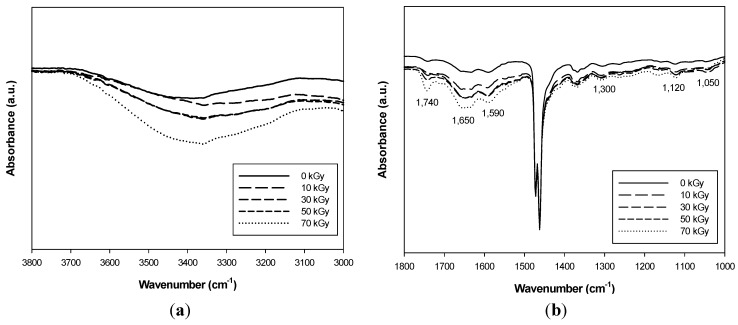
Fourier transform infrared spectroscopy (FT-IR) spectra of PU (1 phr)/HDPE composite at various absorption doses. (**a**) Wavenumber range 3800–3000 cm^−1^; (**b**) wavenumber range 1800–1000 cm^−1^.

Figure 3 shows the FT-IR spectra of the PU/HDPE composite at various absorption doses in the range of 1000 to 3800 cm^−1^. As shown in the PU/HDPE composite spectra, a new sharp peak of a typical non-hydrogen-bonded urethanecarbonyl group was observed at 1740 cm^−1^. In addition, the stretching vibration of the C=O bond of the urea groups at 1650 cm^−1^ and the NH_2_ scissoring vibration of primary amines at 1590 cm^−1^ were observed [16]. The stretching vibration peaks of the NH groups of the urea groups occurred in the region of 3200–3400 cm^−1^ [17]. The absorbance intensity of these peaks increased with increasing radiation doses. This shows that the urea group of the PU was grafted with the PE-g-MA as a compatibilizer on the surface of the HDPE due to the radiation-induced grafting. In addition, the PU signal in the PU/HDPE composites revealed characteristic bands at 1250 cm^−1^ (C–N stretching), 1120 cm^−1^ (C=O stretching and O–CH_2_ stretching) and 1050 cm^−1^ (C–O stretching) [18]. This result indicated that the PU was easily introduced to the HDPE during the melt processing in the blender after the successful radiation-induced grafting of the PU with PE-g-MA.

**Figure 4:**
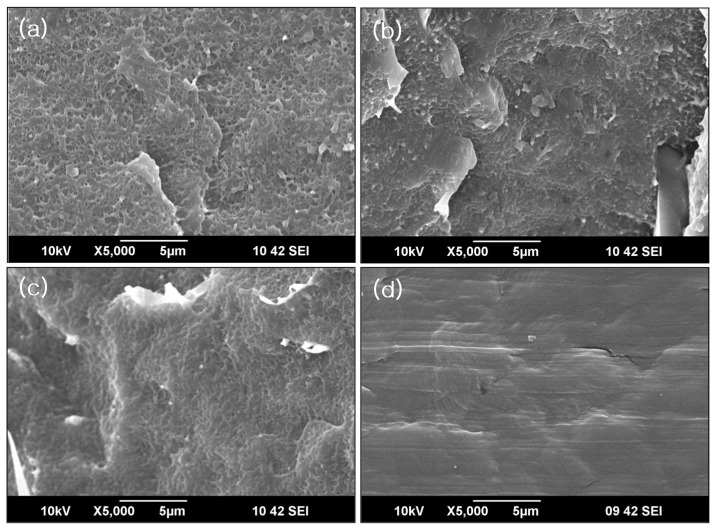
Scanning electron microscope (SEM) micrographs of PU (1 phr)/HDPE composites at various absorption doses. (**a**) 0 kGy; (**b**) 30 kGy; (**c**) 50 kGy; (**d**) 70 kGy.

The SEM micrographs provided further evidence for the success of the radiation-induced grafting. The surface morphologies of the PU/HDPE composites at various absorption doses are presented in Figure 4. The characteristic morphology of the PU/HDPE composites depended on the radiation dose. The white domains of the PU in the HDPE matrix at 0 kGy were irregularly distributed due to weak interfacial bonding. However, the white domains of the PU in the HDPE matrix gradually disappeared for increasing radiation doses. When the absorbed dose was 70 kGy, the surface morphologies were almost smooth and presented a single phase. This result indicated that a higher irradiation dose promoted interfacial adhesion between the PU domain and the HDPE matrix. This result is probably that the improved interfacial adhesion was attributed to the strong chemical interaction between the PU and the PE-g-MA and the strong physical interaction between the HDPE and the PE-g-MA. The grafting of PE-g-MA onto PU surface was firstly performed by electron beam irradiation. The chemical interaction presumably resulted from reaction between the PU and maleic anhydride in PE-g-MA during radiation treatment. The PE-g-MA reacts easily with the PU according to the increasing radiation dose and was located at the interface between the PU and the HDPE during the melt processing in the blender, which improved the interfacial interactions and the mechanical properties of the resultant blends [15,19,20].

**Figure 5:**
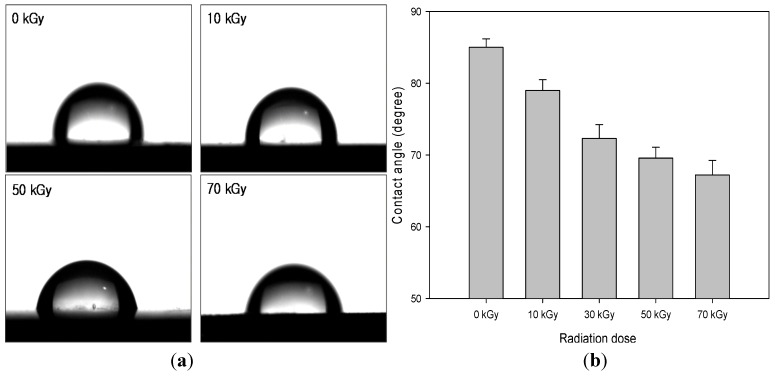
Contact angle of PU (1 phr)/HDPE composites at various absorption doses. (**a**) Water contact angle images; (**b**) water contact angle values.

Comparison of the water contact angles of the blends can reveal changes in the hydrophilic characterization of the blends depending on the PU that was introduced to the HDPE after the radiation-induced grafting. Figure 5 shows the water contact angles of the PU/HDPE composites at various absorption doses. The water contact angle decreased for increasing the radiation doses. As shown in Figure 5, the water contact angle at 0 kGy was 84°, whereas at 70 kGy, it decreased to 67°. It can be attributed to the higher hydrophilicity of the PU. Namely, the improved interfacial adhesion between the PU domain and the HDPE matrix due to the radiation-induced grafting of PU with PE-g-MA led to enhanced surface wettability [9].

**Figure 6:**
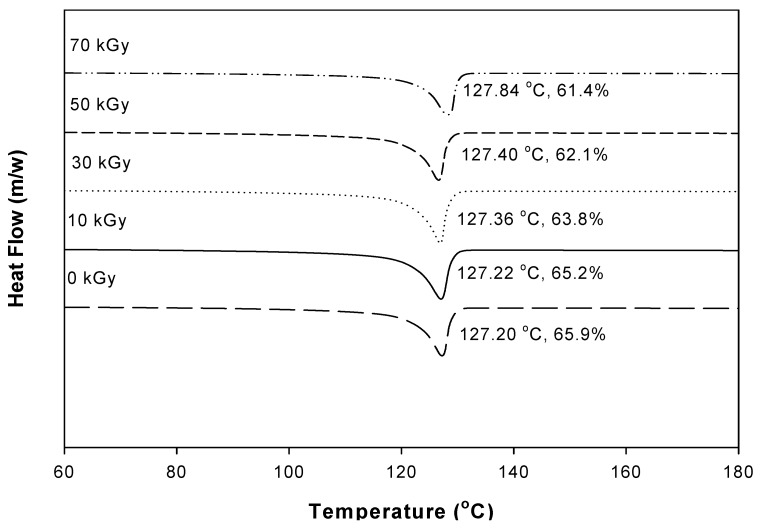
Differential scanning calorimetry (DSC) micrographs of PU (1 phr)/HDPE composites at various absorption doses.

Figure 6 shows the DSC heating thermograms of the PU/HDPE composites at various absorption doses under a heating rate of 10 °C/min. The melting peak at approximately 127°–128° corresponds to the melting temperature of the HDPE. A slight increase in the melting temperature was observed for the PU/HDPE composites with increasing radiation doses. However, the crystallinity decreased with increasing radiation dose. It can be explained that the regularity of the crystallization of HDPE was disturbed by the grafted side chains. Thus, the irregular structures formed through the grafting process reduce the crystallinity of HDPE molecular chains [21].

**Figure 7:**
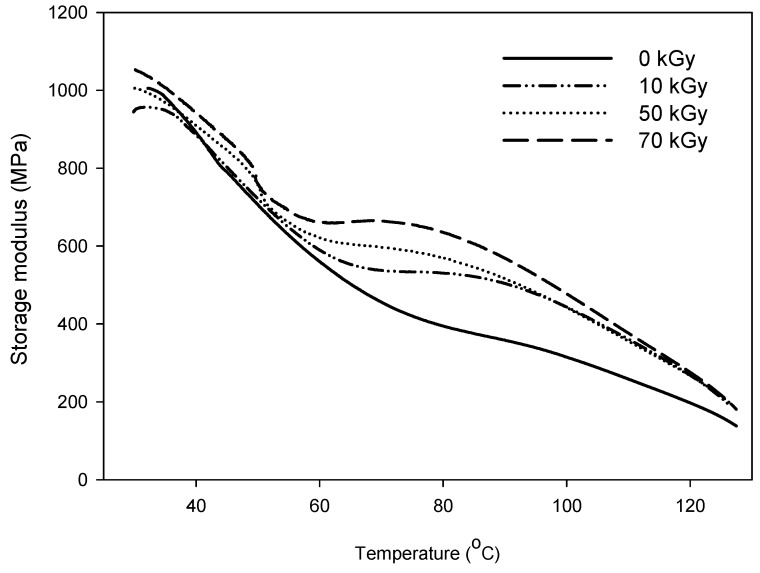
Storage modulus PU (1 phr)/HDPE composite at various absorption doses.

The thermal-mechanical properties of the samples were measured using a DMA, which was varied from room temperature to 130 °C at a heating rate of 5 °C/min. The temperature dependencies of the storage modulus of the PU/HDPE composites with increasing radiation doses are shown in Figure 7. The non-irradiated composites exhibited the lowest storage moduli in this study. Electron beam irradiation induced an increase in the storage modulus of the composites. This result is similar to the tensile strength trend shown in Figure 1, which may be because the PU/HDPE composite exhibits strong interfacial bonding between the PU and the HDPE due to the radiation-induced grafting. 

## 4. Conclusions

In this study, we developed a recycling method for waste PU that utilizes the radiation grafting. It is well known that PU/HDPE composites exhibit poor mechanical properties, such as tensile strength and elongation at break, due to their incompatibility. However, the radiation grafting on PU/HDPE composites has enabled for developing composites with better properties.

PU was easily introduced to HDPE via melt processing in a blender after radiation-induced grafting of PU with PE-g-MA. The PE-g-MA easily reacted with the PU according to increasing radiation doses and was located at the interface between the PU and the HDPE during the melt processing in the blender, which improved the interfacial interactions and the mechanical properties of the resultant composites. However, the elongation at break for PU contents >2 phr drastically decreased. When the PU content in the HDPE composites was >2 phr, the compatibility between the PU and the HDPE drastically deteriorated, and incompatibility was observed even though PU and PE-g-MA underwent electron beam irradiation. Storage modulus PU (1 phr)/HDPE composite at various absorption doses.
